# Correction: Antimicrobial and alpha-glucosidase inhibitory flavonoid glycosides from the plant *Mussaenda recurvata*: *in vitro* and *in silico* approaches

**DOI:** 10.1039/d4ra90031f

**Published:** 2024-04-02

**Authors:** Tran Thi Ngoc Mai, Phan Nhat Minh, Nguyen Tan Phat, Thuc Huy Duong, Tran Nguyen Minh An, Van Son Dang, Nguyen Van Hue, Mai Dinh Tri

**Affiliations:** a Institute of Applied Sciences, HUTECH University 475A Dien Bien Phu Street, Ward 25, Binh Thanh District Ho Chi Minh City Vietnam; b Graduate University of Science and Technology, Vietnam Academy of Science and Technology 18 Hoang Quoc Viet, Cau Giay Hanoi Vietnam maidinhtri@gmail.com; c Institute of Chemical Technology, Vietnam Academy of Science and Technology 1A TL29 Street, Thanh Loc Ward, District 12 Ho Chi Minh City Vietnam; d Department of Chemistry, Ho Chi Minh City University of Education 280 An Duong Vuong Street, District 5 748342 Ho Chi Minh City Vietnam huydt@hcmue.edu.vn; e Faculty of Chemical Engineering, Industrial University of Ho Chi Minh City Ho Chi Minh City 71420 Vietnam trannguyenminhan@iuh.edu.vn; f Institute of Tropical Biology, Vietnam Academy of Science and Technology 85 Tran Quoc Toan Street, District 3 Ho Chi Minh City 700000 Vietnam; g University of Agriculture and Forestry, Hue University 52000 Vietnam

## Abstract

Correction for ‘Antimicrobial and alpha-glucosidase inhibitory flavonoid glycosides from the plant *Mussaenda recurvata*: *in vitro* and *in silico* approaches’ by Tran Thi Ngoc Mai *et al.*, *RSC Adv.*, 2024, **14**, 9326–9338, DOI: https://doi.org/10.1039/D4RA00666F.

The authors would like to clarify the affiliation list of the published version to correctly show where the work was conducted.

In the original article, the affiliations at the time of publication of one author (Van Song Dang) were listed as:

^a^Institute of Applied Sciences, HUTECH University, 475A Dien Bien Phu Street, Ward 25, Binh Thanh District, Ho Chi Minh City, Vietnam

^f^Institute of Tropical Biology, Vietnam Academy of Science and Technology, 85 Tran Quoc Toan Street, District 3, Ho Chi Minh City, Vietnam.

However, the work was conducted at:

^b^Graduate University of Science and Technology, Vietnam Academy of Science and Technology, 18 Hoang Quoc Viet, Cau Giay, Hanoi, Vietnam

^f^Institute of Tropical Biology, Vietnam Academy of Science and Technology, 85 Tran Quoc Toan Street, District 3, Ho Chi Minh City, Vietnam.

The corrected list of affiliations at the time of publication of the original article is presented here.

The Royal Society of Chemistry apologises for these errors and any consequent inconvenience to authors and readers.

## Supplementary Material

